# Limited clinical benefit of medial meniscus posterior root repair combined with high tibial osteotomy in varus knee osteoarthritis: A systematic review and meta‐analysis

**DOI:** 10.1002/jeo2.70431

**Published:** 2025-09-22

**Authors:** Gabriele Cortina, Stefano Mauro Antuofermo, Giuseppe Francesco Papalia, Raffaele Cortina, Vincenzo Condello, Simone Perelli, Joan Carles Monllau, Vincenzo Madonna

**Affiliations:** ^1^ Department of Orthopaedic and Trauma Surgery Pierangeli Clinic Pescara Italy; ^2^ Department of Orthopaedic, Joint Prosthetic, Arthroscopic Surgery and Sports Traumatology Humanitas Castelli Bergamo Italy; ^3^ Orthopaedic Department University of Sassari Sassari Italy; ^4^ Oncological Orthopaedics Department IFO – IRCCS Regina Elena National Cancer Institute Rome Italy; ^5^ Institut Català de Traumatologia i Medicina de l'Esport (ICATME), Hospital Universitari Dexeus Universitat Autònoma de Barcelona Barcelona Spain; ^6^ Department of Surgery and Morphologic Science, Orthopaedic Surgery Service Universitat Pompeu Fabra, Hospital del Mar Barcelona Spain

**Keywords:** high tibial osteotomy (HTO), medial meniscus posterior root tear (MMPRT), meniscal extrusion, meniscal repair, osteoarthritis

## Abstract

**Purpose:**

Medial meniscus posterior root tears (MMPRTs) are biomechanically comparable to total meniscectomy, leading to meniscal extrusion, increased tibiofemoral contact pressure and accelerated osteoarthritis (OA) in varus‐aligned knees. While high tibial osteotomy (HTO) is effective in unloading the medial compartment, the added value of repairing the MMPRT during HTO remains debated. This systematic review and meta‐analysis aimed to evaluate whether combined MMPRT repair and HTO provide superior short‐term clinical and radiological outcomes compared to HTO alone.

**Methods:**

A systematic search of PubMed, Cochrane and Scopus was performed in March 2025. Comparative studies evaluating HTO with or without concurrent MMPR repair in patients with varus knee and medial OA were included. Primary outcomes were clinical scores (International Knee Documentation Committee [IKDC], Lysholm, Knee Society Score [KSS] and Hospital for Special Surgery [HSS]), radiographic parameters (joint line convergence angle [JLCA], hip–knee–ankle [HKA] angle and joint space width), meniscal extrusion and second‐look arthroscopic findings. Statistical analysis was conducted using a random‐effects model with Review Manager 5.4.

**Results:**

Eight retrospective comparative studies (*n* = 630 patients) met the inclusion criteria. MMPRT repair plus HTO demonstrated statistically higher IKDC scores (MD = 3.56; *p* = 0.001) compared to HTO alone; however, there were no significant differences between groups in terms of Lysholm, KSS function and HSS scores. Radiographically, minimal improvements were noted in JLCA (MD = −0.25; *p* = 0.006), without clear clinical implications. Meniscal extrusion did not differ significantly between groups (MD = 0.30; *p* = 0.72). Second‐look arthroscopy revealed complete root healing in 22% of cases. The risk of bias was moderate to high.

**Conclusion:**

Short‐term follow‐up shows that combining MMPRT repair with HTO yields statistically better IKDC clinical scores. Furthermore, the actual benefit of combining MMPRT repair with HTO in routine clinical practice is questionable. Prospective studies with longer follow‐up are required to clarify the long‐term clinical impact of MM.

**Level of Evidence:**

Level III, systematic review and meta‐analysis.

**Abbreviations**
BMI: body mass index
CA: contact area
CP: contact pressure
HKA: hip–knee–ankle
HSS: Hospital for Special Surgery
HTT: high tibial osteotomy
IKDC: International Knee Documentation Committee
IQR: interquartile range
JLCA: joint line convergence angle
JSW: joint space width
KSS: Knee Society Score
MD: mean difference
MMPRT: medial meniscus posterior root tear
MRI: magnetic resonance imaging
OA: osteoarthritis
OWHTO: open‐wedge high tibial osteotomy
PRISMA: Preferred Reporting Items for Systematic Reviews and Meta‐Analyses
ROBINS‐I: Risk of Bias In Non‐Randomised Studies ‐ of Interventions
SD: standard deviation
TKA: total knee arthroplasty

## INTRODUCTION

Medial meniscus posterior root tears (MMPRTs) are biomechanically equivalent to a total meniscectomy, disrupting hoop stress and increasing tibiofemoral contact pressure (CP), thereby accelerating degeneration of the medial compartment [1, 31]. This pathology, often described as a ‘silent epidemic’, is increasingly recognised as a major driver of osteoarthritis (OA) progression, particularly among middle‐aged individuals with varus malalignment [7].

In varus‐aligned knees, MMPRTs worsen medial compartment overload, leading to meniscal extrusion and a 25%–40% rise in medial compartment CPs, triggering a cascade of cartilage degradation [3, 21]. Without intervention, MMPRTs frequently progress to end‐stage OA, with reported total knee arthroplasty (TKA) rates of 32%–45% within 5 years [6, 20].

Surgical management has evolved, with transtibial pull‐out repair considered the gold standard for MMPRT [4, 9]. This technique can restore 70%–80% of native CPs and reduce extrusion by 50%–60%, although healing remains incomplete in a substantial proportion of cases [10, 26]. Despite these advances, isolated repairs do not address biomechanical overload in varus knees, resulting in persistent medial meniscus extrusion and OA progression and highlighting the need for concurrent alignment correction [2, 16]. In addition, opening‐wedge high tibial osteotomy (OWHTO) is a widely accepted treatment for patients with OA and varus alignment, aiming to offload the medial compartment and postpone the need for TKA [23]. Given the frequent coexistence of MMPRTs and varus malalignment, combining MMPRT repair with OWHTO may enhance joint preservation [11].

A prior meta‐analysis by Kyun‐Ho et al. and a systematic review by Wang et al. evaluated the effect of MMPRT repair and OWHTO in medial varus‐aligned knee OA and reported improved healing and some clinical benefits with combined procedures [24, 41]. However, both reviews included heterogeneous study designs and lacked consistent control groups, limiting the strength of their conclusions. The aim of this systematic review and meta‐analysis was to evaluate whether combining MMPRT repair with HTO results in superior clinical and radiological outcomes compared to HTO alone in patients with varus knee and medial OA. The authors hypothesised that the combined procedure would yield significantly better outcomes than HTO alone.

## MATERIALS AND METHODS

This systematic review with meta‐analysis was conducted and reported according to the Preferred Reporting Items for Systematic Reviews and Meta‐Analysis (PRISMA) guidelines and the Cochrane Handbook of Systematic Reviews of Interventions recommendations.

### Search strategy

Two authors (G.C. and S.M.A) systematically searched PubMed, Cochrane Central and Scopus in March 10, 2025, using the following search string: (((posterior medial meniscus root OR MMPR OR medial meniscus root tear) AND (osteotomy OR osteotomies OR proximal tibial osteotomy OR high tibial osteotomy OR HTO) AND (root repair OR meniscal repair OR suture anchor repair OR pullout repair OR pullout trans‐tibial repair))). No filters or date/language restrictions were applied to the search strategy. Additionally, backwards snowballing was performed by screening the reference lists of relevant review articles and included studies to identify additional eligible studies.

### Eligibility criteria and study selection

Two independent reviewers (G.C. and S.M.A.) screened all the titles and abstracts. Full‐text screening was done on articles that showed discrepancies. Suitable studies were selected based on the following inclusion criteria: (1) prospective and retrospective comparative studies; (2) reporting clinical, radiological or second‐look arthroscopic outcomes and (3) of HTO with concurrent MMPRT repair in patients with radiograph findings of medial OA and MRI or arthroscopic findings of MMPRTs. The exclusion criteria were as follows: (1) review/technical papers, (2) inaccessible data or full‐text, (3) not written in English, (4) studies without a control group (HTO alone) and (5) studies with a follow‐up less than 12 months. A third author then reviewed the selection studies, and a discussion resolved discrepancies. In all included studies, the diagnosis of MMPRT was based on preoperative MRI and/or arthroscopic findings. In the HTO‐alone control groups, patients with MRI or intraoperative arthroscopic evidence of MMPRT were excluded, ensuring a valid comparison with those undergoing combined repair.

### Data extraction and endpoints

Each study was evaluated according to its inclusion criteria. After deleting the duplicates, the relevant full‐text articles from the electronic search were obtained and evaluated. The bibliography of each study was manually searched to find any potentially relevant papers. Finally, the two reviewers (G.C. and S.M.A.) examined the remaining manuscripts to select the included studies. Two authors (G.F.P. and G.C.) extracted these data from each paper: authors, type of study with level of evidence, number of patients in the MMPRT repair + HTO group and HTO alone group, follow‐up, mean age and gender, BMI, type of HTO implant, sequence of surgery, type of osteotomy, target alignment and post‐operative clinical, radiological and second‐look arthroscopic outcomes. One of the included studies reported data as mean with interquartile range (IQR) rather than standard deviation (SD) [8]; therefore, a validated converter was used to transform the IQR into SD to properly conduct the statistical analysis [30, 40].

### Quality assessment of included studies

The ROBINS‐I tool (“Risk of Bias In Non‐randomised Studies of Interventions”) was used to rate the quality of studies included in the current systematic review and meta‐analysis. Two authors (G.C. and S.M.A.) independently evaluated the quality of each study.

### Statistical analysis and evidence synthesis

A random‐effects meta‐analysis was conducted using study‐level, publicly available data. Means and SDs were pooled to calculate mean differences (MDs) and 95% confidence intervals (CIs) for continuous endpoints. Statistical analyses were conducted using Review Manager software version 5.4.1 (Cochrane Collaboration). *p* values less than 0.05 were deemed significant for treatment effects. Cochran's *Q* test and *I* ^2^ were used to assess between‐study heterogeneity; *p* values less than 0.10 and *I* ^2^ ≥ 50% were considered significant for heterogeneity.

## RESULTS

### Study characteristics

**Figure 1:**
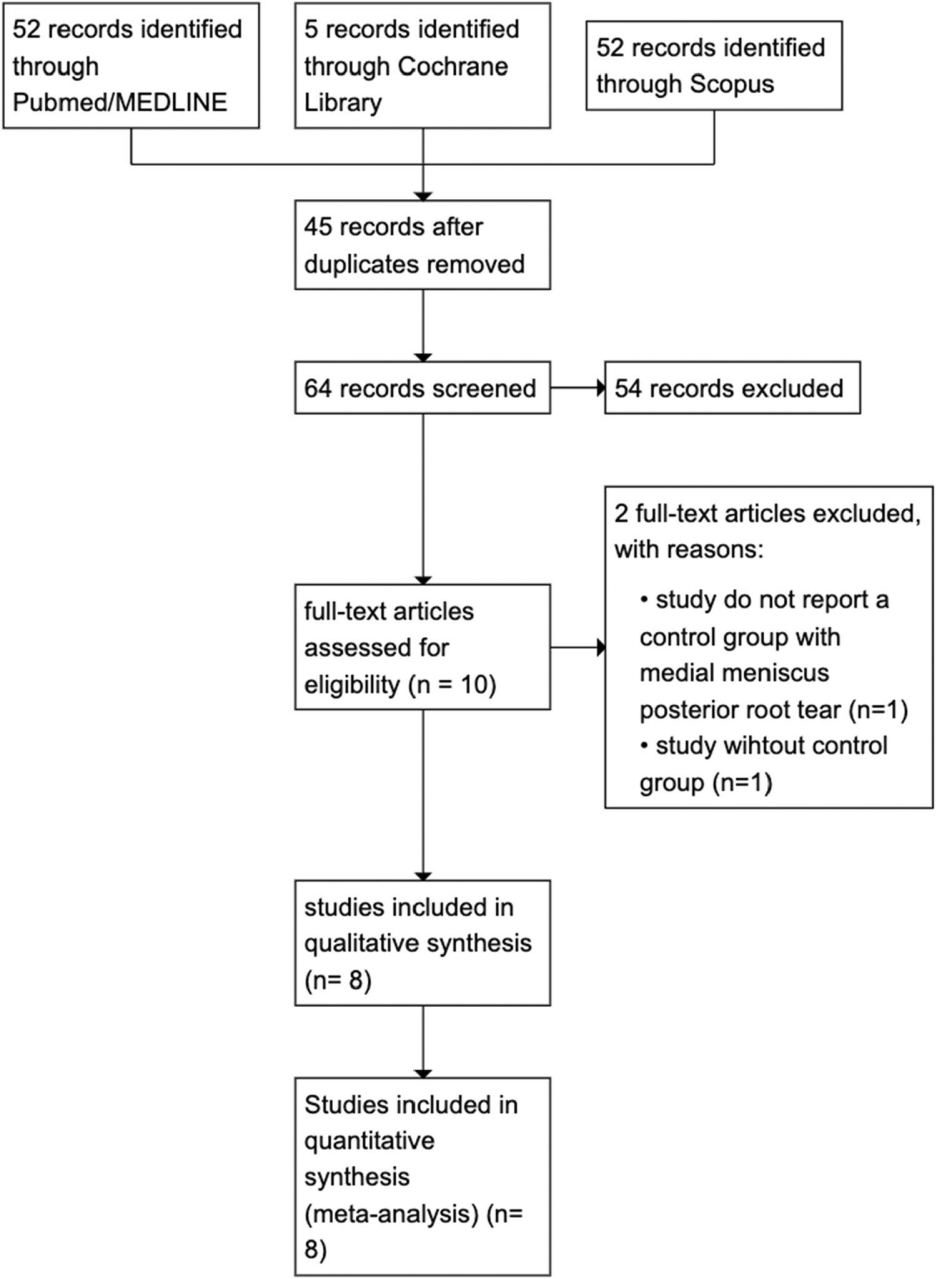
PRISMA flowchart. PRISMA, Preferred Reporting Items for Systematic Reviews and Meta‐analyses.

As illustrated in Figure 1, the search strategy yielded 109 abstracts or manuscripts. Forty‐five records were removed because of duplicates. The 64 remaining studies were thoroughly reviewed. Of those, 56 were either unrelated to the topic or had one or more exclusion criteria. Eight published manuscripts, all retrospective comparative studies, met all inclusion criteria. A non‐overlapping population of 630 patients was included, of whom 300 knees (50.2%) underwent MMPRT repair + HTO, while 298 (49.83%) underwent HTO alone. The mean follow‐up across studies ranged from 24 to 28 months, limiting all interpretations to the short‐term effectiveness of MMPRT repair in conjunction with HTO. Characteristics of the included studies are summarised in Table 1.

**Table 1:** Design and characteristics studies included in the meta‐analysis.

	Choi Y.S. (2023) (No. of pt = 80 pt)		Guo H. (2023) (No. of pt = 153 pt)		Ke X. (2021) (No. of pt = 100)		Lee D.W. (2020) (No. of pt = 71)			Lee S. (2019) (No. of pt = 57)		Suh D.W. (2020) (No. of pt = 81)		Lan M. (2024) (No. of pt = 54)		Dastan A. E. (2025) (No. of pt = 34)	
Characteristics	MMPRT repair + HTO (no. of Knees = 40)	HTO (*n* = 40)	MMPRT repair + HTO (*n* = 73)	HTO (*n* = 80)	MMPRT repair + HTO (*n* = 30)	HTO (*n* = 36)	Trans‐tibial pull‐out MMPRT repair + HTO (*n* = 25)	MMPRT all‑inside repair + HTO (*n* = 24)	HTO (*n* = 22)	MMPRT repair + HTO (*n* = 25)	HTO (*n* = 32)	MMPRT repair + HTO (*n* = 43)	HTO (*n* = 38)	MMPRT repair + HTO (*n* = 21)	HTO (*n* = 33)	MMPRT repair + HTO (*n* = 19)	HTO (*n* = 17)
Study design	Retrospective cohort		Retrospective cohort		Retrospective cohort		Retrospective cohort			Retrospective cohort		Retrospective cohort		Retrospective cohort		Retrospective cohort	
Mean follow‐up, in months (range)	14.9 (12–16)		30.7 (3.5)	29.4 (2.9)	29.0 (3.2)	30.4 (3.0)	24	26	28.5 (5.7)	24	26	24		30.7 (6.9)	29.9 (5.1)	30	28
Mean age, years	59.4	59.8	41.0 (6.0)	42.8 (5.1)	55.4 (7.2)	55.2 (7.9)	59.8 (5.3)	57.4 (5.8)	56.5 (5.3)	58.1 (4.2)	59.8 (5.3)	55.7 (5.6)	56.2 (4.1)	51.5 (6.4)	51.0 (7.4)	52	54
Gender female (male)	35 (5)	32 (8)	10 (63)	14 (66)	26 (4)	26 (8)	24 (10)	23 (1)	20 (2)	18 (8)	24 (10)	35 (8)	30 (8)	10 (11)	10 (23)	19	15 (2)
BMI (range)	26.4 (3.0)	27.6 (3.5)	22.5 (3.3)	24.4 (2.3)	23.1 (2.9)	23.8 (2.8)	26.8 (3.7)	26.1 (2.4)	25.2 (2.8)	27.1 (3.2)	26.8 (3.7)	26.9 (4.2)	26.1 (3.2)	23.4 (3.1)	22.2 (4.1)	32	31
HTO implants	TomoFix plate (DePuy Synthes)		Locking plate (Dabo Medical Technology Co., Ltd.)		TomoFix plate (DePuy Synthes)		Wedge locking plate (TDM)			Locking plate (DWLP, TDM)		The OhtoFix type I plate (Ohtomedical Co., Ltd.)		TomoFix plate (DePuy Synthes)		TomoFix plate (DePuy Synthes)	
Sequence of surgery	1° MMPRT repair 2° OWHTO		1° OWHTO 2° MMPRT repair		1° MMPRT repair 2° OWHTO		1° MMPRT repair 2° OWHTO			NR		1° MMPRT repair 2° OWHTO		1° MMPRT reconstruction 2° OWHTO		1° MMPRT reconstruction 2° OWHTO	
Type of osteotomy	Biplanar		Biplanar		Biplanar		Biplanar			Biplanar		Uniplanar or biplanar		Biplanar		Uniplanar	
Target alignment of HTO	Fujisawa point		Fujisawa point		Fujisawa point		Fujisawa point			Fujisawa point		Fujisawa point		Fujisawa point		Fujisawa point	
Second‐look arthroscopy	Yes		No		Yes		Yes			Yes		No		Yes		No	

Abbreviations: BMI, body mass index; HTO, high tibial osteotomy; MMPRT, medial meniscus posterior root tear; OWHTO, open wedge high tibial osteotomy.

### Clinical outcomes

**Figure 2:**
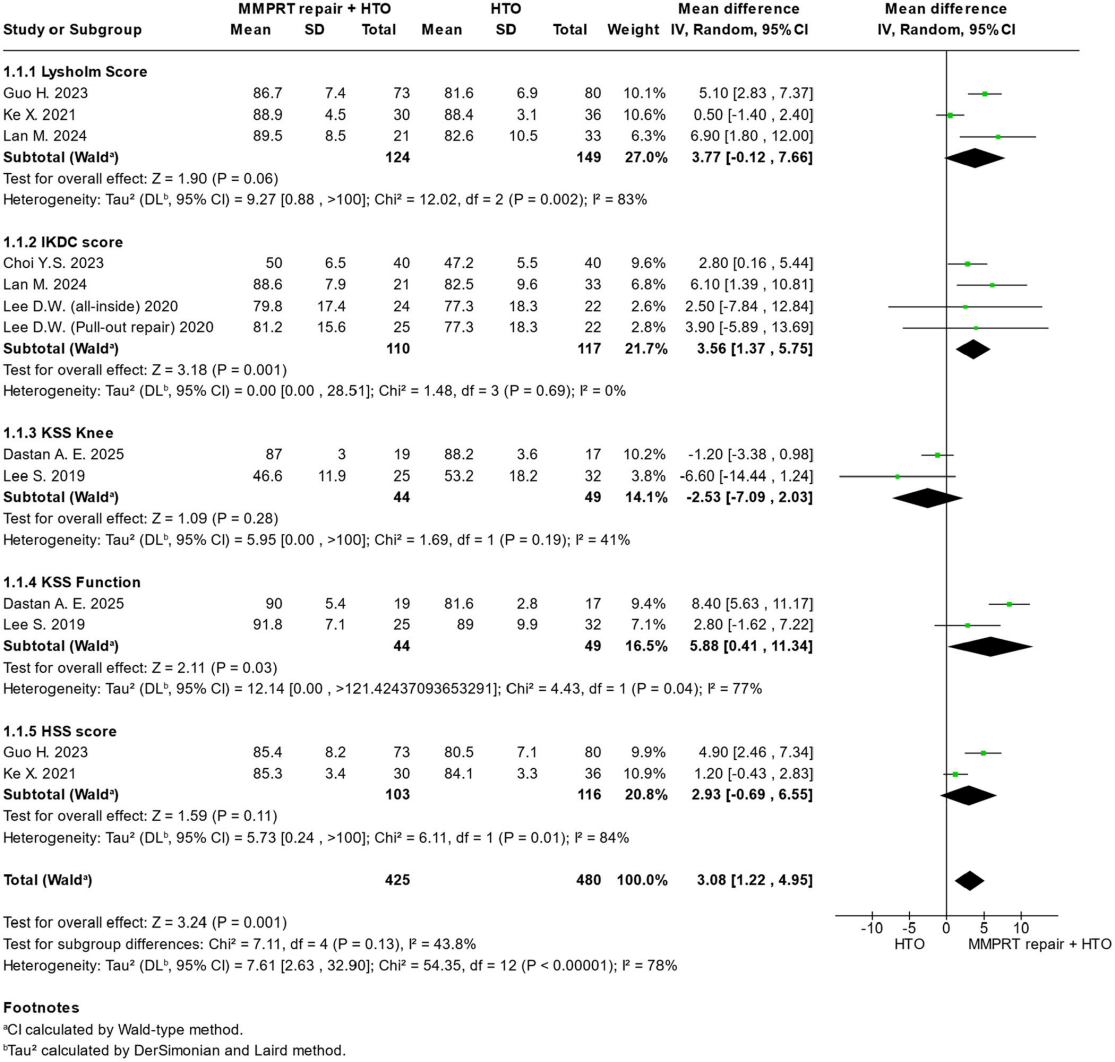
Forest plots illustrating the treatment effect on clinical outcomes between MMPRT repair + HTO and HTO alone for the Lysholm score, IKDC score, KSS Knee score, KSS Function score and HSS score. CI, confidence interval; HSS, Hospital for Special Surgery; HTO, high tibial osteotomy; IKDC, International Knee Documentation Committee; KSS, Knee Society Score; MMPRT, medial meniscal posterior root tear; SD, standard deviation.

**Figure 3:**
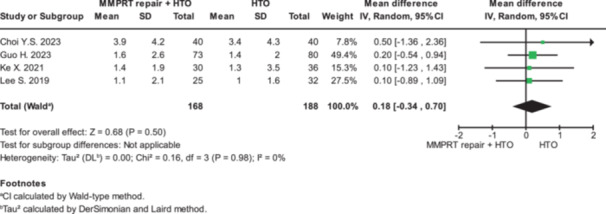
Forest plot illustrating the treatment effect on flexion contracture between MMPRT repair + HTO and HTO alone. CI, confidence interval; HTO, high tibial osteotomy; MMPRT, medial meniscus posterior root tear; SD, standard deviation.

The clinical outcomes are summarised in Figures 2 and 3 and Table 2. Relative to HTO alone, MMPRT repair plus HTO was associated with a higher International Knee Documentation Committee (IKDC) score (MD = 3.56; 95% CI = 1.37–5.75; *p* = 0.001). There was no significant difference between MMPRT repair plus HTO as compared with HTO alone in terms of Lysholm Knee Scoring Scale (MD = 3.77 points; 95% CI = −0.12 to 7.66; *p* = 0.06), Knee Society Score (KSS) function (MD = 5.88 points; 95% CI = 0.41–11.34; *p* = 0.09), Hospital for Special Surgery (HSS) (MD = 2.93, 95% CI = −0.69 to 6.55; *p* = 0.11), KSS Knee (MD = −2.53, 95% CI = −7.09 to 2.03; *p* = 0.28) or flexion contracture (MD = 0.02; 95% CI = −0.50 to 0.54; *p* = 0.94). Overall, clinical outcomes presented statistically significant differences in favour of MMPRT repair + HTO group (MD = 3.10, 95% CI = 1.41–4.79; *p* = 0.0003).

**Table 2:** Clinical and radiological outcomes of the included studies.

	Choi Y.S. (2023) (No. of pt = 80 pt)		Guo H. (2023) (No. of pt = 153 pt)		Ke X. (2021) (No. of pt = 100)		Lee D.W. (2020) (No. of pt = 71)			Lee S. (2019) (No. of pt = 57)		Suh D.W. (2020) (No. of pt = 81)		Lan M. (2024) (No. of pt = 54)		Dastan A. E. (2025) (No. of pt = 34)	
Parameters	MMPRT repair + HTO (no. of Knees = 40)	HTO (*n* = 40)	MMPRT repair + HTO (*n* = 73)	HTO (*n* = 80)	MMPRT repair + HTO (*n* = 30)	HTO (*n* = 36)	Trans‐tibial pull‐out MMPRT repair + HTO (*n* = 25)	MMPRT all‑inside repair + HTO (*n* = 24)	HTO (*n* = 22)	MMPRT repair + HTO (*n* = 25)	HTO (*n* = 32)	MMPRT repair +HTO (*n* = 43)	HTO (*n* = 38)	MMPRT repair +HTO (*n* = 21)	HTO (*n* = 33)	MMPRT repair +HTO (*n* = 19)	HTO (*n* = 17)
HKA (degree)	182.4 (2.6)	182 (3.5)	181.1 (2.7)	180.1 (1.5)	183.9 (0.9)	183.8 (1.1)	182.2 (1.5)	182.3 (1.7)	182.5 (1.3)	181.9 (1.2)	181 (1.4)	180.9 (2.0)	181.3 (1.8)	180.8 (1.8)	181.3 (1.6)		
MPTA (degree)			90.1 (1.8)	89.2 (2.2)	93.3 (2.5)	94.1 (1.8)											
JLCA (degree)	1.8 (1.4)	2.6 (1.9)	1.9 (0.7)	2.1 (0.7)	1.9 (0.9)	2.2 (0.7)				2.4 (1.5)	2.5 (1.2)						
JSW (mm)										3.7 (1.2)	3.8 (1.3)	3.5 (1.0)	3.6 (1.2)				
IKCD	50 (6.5)	47.2 (5.5)					81.2 (15.6)	79.8 (17.4)	77.3 (18.3)					88.6 (7.9)	82.5 (9.6)		
Lysholm score			86.7 (7.4)	81.6 (6.9)	88.9 (4.5)	88.4 (3.1)								89.5 (8.5)	82.6 (10.5)		
HSS score			85.4 (8.2)	80.5 (7.1)	85.3 (3.4)	84.1 (3.3)											
Tegner score			6 (2‐8)	5 (2–8)			5.2 (1.2)	4.9 (1.0)	5.1 (1.2)								
WOMAC score (total)										6.4 (5.5)	9.2 (5.3)	77 (11)	76 (11)				
KS knee										46.6 (11.9)	53.2 (18.2)					87 (3)	88.2 (3.6)
KS function score										91.8 (7.1)	89.0 (9.9)					90 (5.4)	81.6 (2.8)
Flexion contracture (degree)	3.9 (4.2)	3.4 (4.3)	1.6 (2.6)	1.4 (2.0)	1.4 (1.9)	1.3 (3.5)				1.1 (2.1)	1.0 (1.6)						

*Note*: Results are expressed as means (standard deviation).

Abbreviations: HKA, hip–knee–angle; HSS, Hospital for Special Surgery; HTO, high tibial osteotomy; IKDC, International Knee Documentation Committee; JLCA, joint line convergence angle; JSW, joint space width; KSS, Knee Society Score; MMPRT, medial meniscus posterior root tear; MPTA, medial proximal tibial angle.

### Radiological outcomes

**Figure 4:**
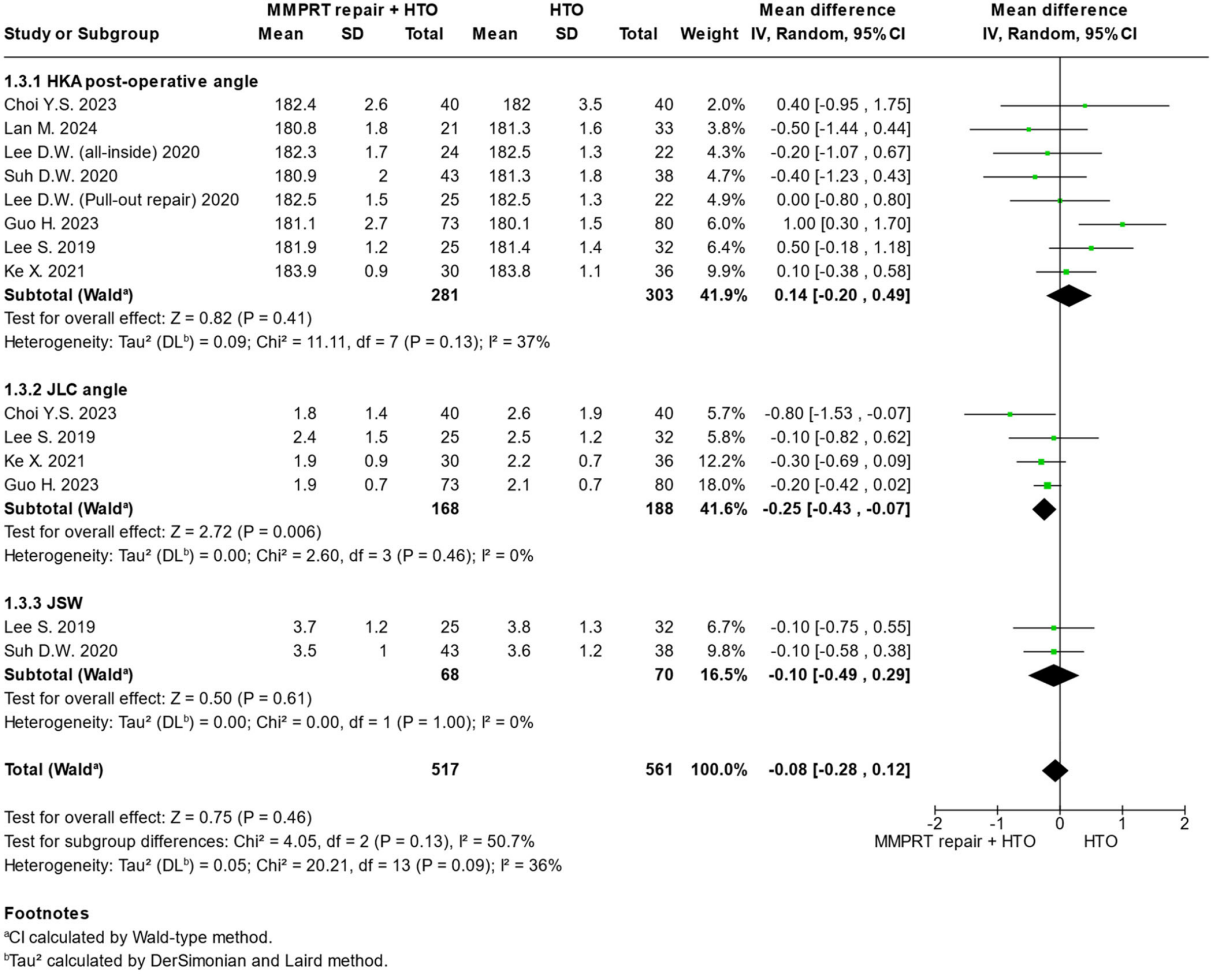
Forest plots illustrating the treatment effect on radiological outcomes between MMPRT repair + HTO and HTO alone. CI, confidence interval; HKA, hip–knee–ankle; HTO, high tibial osteotomy; JLC, joint line convergence; MMPRT, medial meniscus posterior root tear; SD, standard deviation.

The radiological outcomes are summarised in Figure 4 and Table 2. MMPRT plus HTO was associated with significantly better JLC angle (MD = −0.25; 95% CI = −0.43 to −0.07; *p* = 0.006), post‐operative HKA angle (MD = 0.14; 95% CI = −0.20 to 0.49; *p* = 0.41) and joint space width (MD = −0.10; 95% CI = −0.49 to 0.29; *p* = 0.61).

### Post‐operative meniscal extrusion

**Figure 5:**
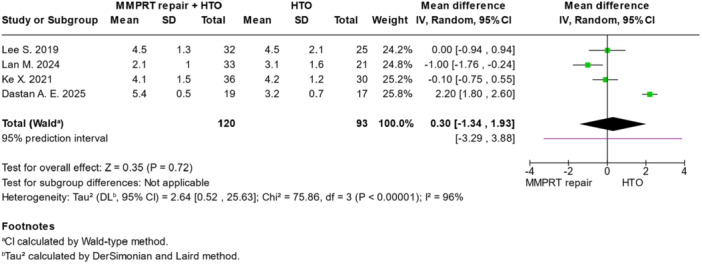
Forest plots illustrating the treatment effect on meniscal extrusion between MMPRT repair + HTO and HTO alone. CI, confidence interval; HTO, high tibial osteotomy; MMPRT, medial meniscus posterior root tear; SD, standard deviation.

Post‐operative meniscal extrusion was analysed in three studies and showed no significant difference between groups (MD = 0.30, 95% CI = −1.34 to 1.93; *p* = 0.72) (Figure 5 and Table 3).

**Table 3:** Medial meniscal extrusion and arthroscopic findings of the included studies.

	Choi Y.S. (2023) (No. of pt = 80 pt)		Ke X. (2021) (No. of pt = 100)		Lee D.W. (2020) (No. of pt = 71)			Lee S. (2019) (No. of pt = 57)		Lan M. (2024) (No. of pt = 54)		Dastan A. E. (2025) (No. of pt = 34)	
Parameters	MMPRT repair + HTO (no. of Knees = 40)	HTO (*n* = 40)	MMPRT repair + HTO (*n* = 73)	HTO (*n* = 36)	Trans‐tibial pull‐out MMPRT repair + HTO (*n* = 25)	MMPRT all‑inside repair + HTO (*n* = 24)	HTO (*n* = 22)	MMPRT repair + HTO (*n* = 25)	HTO (*n* = 32)	MMPRT repair + HTO (*n* = 21)	HTO (*n* = 33)	MMPRT repair + HTO (*n* = 19)	HTO (*n* = 17)
Meniscal healing (%)	11 (27.5) complete 24 (60) partial 5 (12.5) failed	4 (10) complete 15 (37.5) partial 21 (52.5) failed	7 (23) complete 12 (40) partial 11(36.6) failed	2 (5.8) complete 10 (29.4) partial 22 (64) failed	6 (24) good 11 (44) loose 6 (24) scar 2 (8) failed	3 (12.5) good 9 (37.5) loose 9 (17.5) scar 3 (12.5) failed	0 (0) good 2 (9) loose 7 (31.8) scar tissue 13 (59) failed	10 (40) complete healing 9 (36) partial healing 6 (24) failed	5 (15) complete healing 8 (25) partial healing 19 (59.4) failed	18 (85.7) complete 3 (14.3) loose 0 failed	15 (45.4) complete 9 (27.3) loose 9 (27.3) failed		
Post‐op meniscal extrusion (mm)			4.1 (1.5)	4.2 (1.2)				4.5 (1.3)	4.5 (2.1)	2.1 (1.0)	3.1 (1.6)	5.4 (0.5)	3.2 (0.7)
Articular cartilage healing	Preoperative ICRS grade (1/2/3/4) MFC 0/0/22/18 to 5/4/10/21	Preoperative ICRS grade (1/2/3/4) MFC 0/0/22/18 to 8/12/11/9	Outerbridge grade of medial compartment (1/2/3/4): 0/4/14/12 to 0/8/18/4	Outerbridge grade of medial compartment (1/2/3/4): 0/6/16/12 to 0/10/18/6	Preoperative ICRS grade (1/2/3/4): MFC 0/5/13/7 MTP 3/9/11/2, ICRS regeneration grade (excellent/good/poor) MFC 6/14/5 MTP 3/14/8	Preoperative ICRS grade (1/2/3/4): MFC 0/6/13/5 MTP 3/9/11/1 ICRS regeneration grade (excellent/good/poor) MFC 4/12/8 MTP 2/14/8	Preoperative ICRS grade (1/2/3/4): MFC 0/5/11/6 MTP 2/8/10/2 ICRS regeneration grade (excellent/good/poor) MFC 3/11/8 MTP 2/10/10	ICRS grade of the MFC (0/1/2/3/4): 0/0/4/9/12 to 1/3/6/9/6	ICRS grade of the MFC (0/1/2/3/4): 0/1/8/12/14 to 2/6/9/11/7				

*Note*: Results are expressed as means (standard deviation).

Abbreviations: HTO, high tibial osteotomy; ICRS, International Cartilage Repair Society; MFC, medial femoral condyle; MMPRT, medial meniscus posterior root tear; MTP, medial tibial plateau.

### Meniscal healing

**Figure 6:**
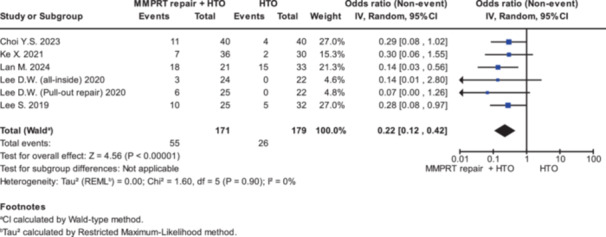
Forest plots illustrating the treatment effect on meniscal healing between MMPRT repair + HTO and HTO alone. CI, confidence interval; HTO, high tibial osteotomy; MMPRT, medial meniscus posterior root tear; SD, standard deviation.

The results of meniscal healing are presented in Table 3 and Figure 6. Six subgroups in five studies reported the second‐look arthroscopic findings on MMPRT healing status [5, 18, 25, 27, 28]. The pooled event rate for complete healing of the medial meniscus posterior root was 0.22 (95% CI = 0.12–0.42; *p* < 0.00001).

### Articular cartilage findings

Second‐look arthroscopic findings of articular cartilage are summarised in Table 3. Four included studies reported the cartilage status evaluated with second‐look arthroscopy [5, 18, 27, 28]. Articular cartilage status was reported using the ICRS grading system in three studies [5, 27, 28] and the Outerbridge grading system in one study [18], both preoperatively and post‐operatively. Moreover, Lee et al. reported cartilage status using both ICRS degeneration and regeneration grading systems [27].

### Quality assessment

**Figure 7:**
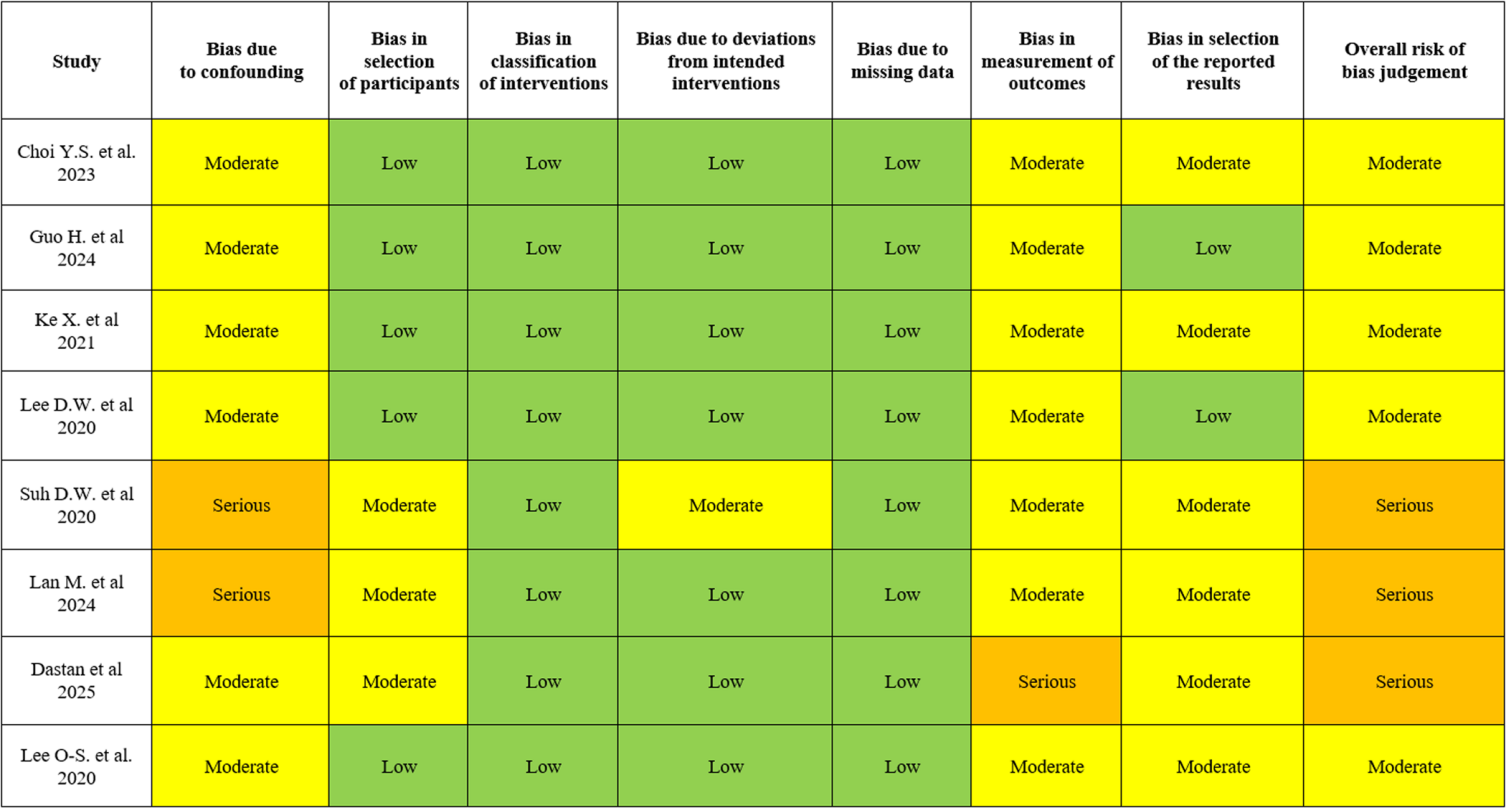
Quality assessment of the study included.

The risk of bias of the included studies was evaluated using the ROBINS‐I tool. Among the included studies, none were classified as having a low risk of bias; five studies presented a moderate risk of bias [5, 14, 18, 27, 28], and three studies were rated as having a serious risk of bias [8, 25, 38]. No studies were judged as having a critical risk. The domains most frequently associated with a moderate or serious risk of bias were confounding and measurement of outcomes. Detailed results for the quality assessment of each included study are reported in Figure 7.

## DISCUSSION

This meta‐analysis evaluated the comparative efficacy of concurrent MMPRT at the time of opening‐wedge HTO versus HTO alone in varus‐aligned knees with early‐to‐moderate medial OA. The findings of this meta‐analysis indicate that the addition of MMPRT repair to HTO is associated with significantly better post‐operative clinical outcomes, as measured by IKDC scores. While the difference in IKDC score reached statistical significance (MD = 3.56 points; *p* = 0.001), the clinical relevance of such a small change remains questionable. Notably, no significant differences were found in other common functional scores such as Lysholm, KSS function or HSS. These findings suggest that the superiority of MMPRT repair in terms of patient‐reported outcomes may be marginal at best. Similarly, the differences observed in radiological outcomes—such as a 0.25° reduction in joint line convergence angle—are minimal and unlikely to translate into meaningful clinical benefit. In addition, no significant differences were found in meniscal extrusion between the groups. Taken together, these observations raise important concerns regarding the actual benefit of combining MMPRT repair with HTO in routine clinical practice.

These data, combined with the fact that three of the included studies were rated as having a serious risk of bias, suggest that the strength of the current evidence is limited. Furthermore, the mean follow‐up of approximately 2 years precludes any inference about long‐term joint preservation or delayed OA progression, which are among the main rationales for performing MMPRT repair during HTO. Therefore, although the concept of combining MMPRT repair with HTO has a strong biomechanical rationale, the clinical benefit of this approach remains uncertain and needs to be validated by future high‐quality prospective trials with longer follow‐up.

Isolated, these procedures may slow medial‐compartment degeneration. A systematic review encompassing 1,086 patients showed conversion‐to‐TKA rates of 11%–54% after partial meniscectomy, up to 35% with conservative treatment and a mere 1% following meniscal repair, underscoring the repair's disease‐modifying potential [37]. Similarly, long‐term follow‐up after HTO demonstrates robust survivorship even in Kellgren–Lawrence (KL) Grade III–IV disease [12]. Coupling these two established strategies appears to improve patient outcomes in varus‐aligned knees with early‐to‐moderate medial OA.

Several systematic reviews have highlighted the positive impact of MMPRT repair alone on clinical and radiological outcomes at over 60 months of follow‐up. However, previous studies have reported conflicting clinical outcomes concerning concurrent MMPRT repair during HTO. The meta‐analyses by Kyun‐Ho et al. [24] and Wang et al. [41] indicated a borderline superiority of concurrent repair mainly due to heterogeneity across studies. Conversely, biomechanical studies consistently advocate for MMPRT repair as essential for restoring joint homoeostasis. Park et al. [34] recently demonstrated that MMPRT repair restores native‐like CPs and contact areas (CAs) in the medial compartment, even following significant valgus correction through HTO. This finding underscores the biomechanical rationale for combining these procedures to enhance medial compartment loading patterns.

Another critical biomechanical aspect is HTO's inadequacy in effectively reducing medial meniscal extrusion (MME) when an MMPRT tear is present [15]. Their cadaveric study illustrated that MME remains unchanged after isolated HTO, while concurrent MMPRT repair significantly reduces extrusion. This emphasises the need for concurrent meniscal treatment with HTO since residual meniscal extrusion strongly correlates with progressive cartilage degeneration and poorer clinical outcomes [22]. These combined biomechanical effects may lead to long‐term clinical benefits by slowing OA progression. However, the clinical significance of minor reductions in extrusion still necessitates further validation through long‐term studies. In fact, the type of medial meniscal lesion, root or not, may not be a marker of poor prognosis [19]. Moreover, Karatekin and Altinayak [17] and Lee et al. [29] demonstrated that post‐operative MME and radiological progression of arthritis did not increase after OWHTO without MMPRT repair. Nevertheless, in the study by Ke et al. [18], even patients with complete healing of the meniscal root might not have improvement in medial meniscus extrusion.

Importantly, executing the two procedures sequentially may be challenging, as there is a considerable risk of the tunnel for the meniscal root converging with the most proximal screws of the osteotomy plate. Moreover, most practitioners prefer to conduct the MMPRT repair first, followed by HTO. In the current meta‐analysis, only Guo and colleagues [14] reported a surgical technique in which HTO was performed first. Regarding the potential overlap between the meniscal root tunnel and proximal plate screws, Nejima et al. [36] demonstrated that fixing the MMPRT in the anteromedial cortex of the tibia resulted in a high incidence of interference between the tibial tunnel and most proximal screws. One alternative would be to use shorter screws to prevent interference, though this might compromise fixation stability in the HTO [35, 39]. Indeed, one approach to mitigate the surgical risk related to tunnel/screw interference may involve modifying the MMPRT repair technique, favouring all‐inside techniques or techniques that utilise suture anchors, which, notably, do not require a tibial tunnel [32, 33, 42].

Nevertheless, the combination of the two surgical procedures does not inherently assure significant clinical improvement. For instance, consider the patients who have received medial meniscus transplantation (MAT) alongside HTO. As shown by a study involving patients who underwent arthroscopic MAT and HTO versus control subjects who received isolated MAT, medial MAT with HTO may yield similar clinical outcomes to those of isolated medial MAT at a mean follow‐up duration of 5.4 years [13].

This review has several limitations. First, the analysis included a limited number of studies, predominantly with short‐term follow‐up and low levels of evidence. Second, significant heterogeneity was observed across studies in terms of design, baseline patient characteristics (e.g., preoperative KL grades) and outcome measures, which may have influenced the results. Third, variations in MMPRT repair techniques among the included studies further contributed to clinical and methodological heterogeneity. Fourth, second‐look arthroscopic findings, such as MMPRT healing status and cartilage regeneration, are subject to interpretation and may introduce bias compared to more objective outcome measures. Finally, the absence of high‐quality randomised controlled trials limits the strength of the conclusions, and definitive recommendations regarding the benefit of concurrent MMPRT repair during HTO cannot yet be made.

The findings of this meta‐analysis have direct relevance for clinical practice. In varus‐aligned knees with early‐to‐moderate medial OA and a MMPRT, performing MMPRT repair in conjunction with HTO appears to yield superior functional outcomes and more favourable radiological parameters compared to HTO alone. This suggests that, when surgically feasible, addressing both the mechanical alignment and the loss of meniscal function can offer synergistic benefits in joint preservation. This means the opportunity to improve patient outcomes by preventing OA progression, reducing pain, and potentially delaying or avoiding TKA. In practical terms, this evidence supports a more comprehensive and biomechanically sound approach to treating this increasingly common clinical scenario, ultimately leading to better long‐term joint health and improved quality of life for patients.

## CONCLUSION

This meta‐analysis of observational studies indicates that, in varus‐aligned knees with early‐to‐moderate medial OA and a MMPRT, performing MMPRT repair with HTO results in statistically superior short‐term clinical outcomes compared to HTO alone. However, the magnitude of these differences appears modest, and their clinical relevance remains uncertain. Radiological differences were minimal, and no significant improvement in meniscal extrusion was observed. Given the moderate to serious risk of bias in the included studies and the short follow‐up periods, the long‐term benefit of this combined surgical approach cannot be determined at this stage. High‐quality randomised controlled trials with extended follow‐up are needed to better define the clinical utility and indications for combining MMPRT repair with HTO.

## AUTHOR CONTRIBUTIONS

Gabriele Cortina conceptualised the study. Gabriele Cortina, Stefano Mauro Antuofermo and Giuseppe Francesco Papalia developed the methodology and conducted the formal analysis. Gabriele Cortina, Stefano Mauro Antuofermo and Giuseppe Francesco Papalia managed software, data curation, visualisation and validation. Gabriele Cortina, Simone Perell, Vincenzo Condello and Raffaele Cortina drafted the original manuscript, while all authors contributed to the review and editing process. Vincenzo Madonna and Joan Carles Monllau supervised the project, administered the research activities and critically reviewed the manuscript.

## CONFLICT OF INTEREST STATEMENT

The authors declare no conflicts of interest.

## ETHICS STATEMENT

The ethics statement is not available.

## Data Availability

Data sharing is not applicable to this article as no data sets were generated or analysed during the current study.
